# High TPX2 expression results in poor prognosis, and Sp1 mediates the coupling of the CX3CR1/CXCL10 chemokine pathway to the PI3K/Akt pathway through targeted inhibition of TPX2 in endometrial cancer

**DOI:** 10.1002/cam4.6958

**Published:** 2024-03-11

**Authors:** Mei Yang, Xiaogang Mao, Lin Li, Jiang Yang, Hui Xing, Chunfan Jiang

**Affiliations:** ^1^ Department of Obstetrics and Gynecology Xiangyang Central Hospital, Affiliated Hospital of Hubei, University of Arts and Science Xiangyang China; ^2^ Institute of Maternity Disease Xiangyang Central Hospital, Affiliated Hospital of Hubei University of Arts and Science Xiangyang China; ^3^ Department of Pathology Xiangyang Central Hospital, Affiliated Hospital of Hubei University of Arts and Science Xiangyang Hubei China

**Keywords:** CX3CR1/CXCL10 chemokine pathway, endometrial cancer, PI3K/Akt signaling pathway, Sp1, TPX2

## Abstract

**Introduction:**

Approximately 30% of individuals with advanced EC have unsatisfactory prognosis. Evidence suggests that TPX2 is frequently upregulated in malignancies and related to cancer progression. Its role and pathological mechanism in EC need further research.

**Methods:**

GSEA and TPX2 expression, GO, KEGG, and prognostic analyses were performed with TCGA data by bioinformatic approaches. Relationships between TPX2 expression and clinicopathological parameters were investigated immunohistochemically and statistically. shRNA and overexpression plasmids were constructed and transfected into AN3CA and Ishikawa cells to evaluate phenotypic changes and injected into nude mouse axillae. Coimmunoprecipitation and chromatin immunoprecipitation were used to identify interacting proteins and promoter‐binding sequences. Changes in TPX2 expression were identified by Western blotting and RT–qPCR.

**Results:**

TPX2 expression was significantly higher in EC tissues than in normal tissues in TCGA and in‐house specimens (all *p* < 0.001). In survival analysis, high TPX2 expression was associated with poor prognosis (*p* = 0.003). TPX2 overexpression stimulated cancer cell proliferation, promoted the G0‐G1‐to‐G2/M transition, enhanced invasion and migration, and accelerated tumor growth in nude mice. TPX2 regulated the CX3CR1/CXCL10 chemokine pathway and activated the PI3K/Akt signaling pathway. Sp1 negatively regulated TPX2 expression, affecting the malignant progression of endometrial cancer cells by coupling the CX3CR1/CXCL10 chemokine pathway to the PI3K/Akt signaling pathway.

**Conclusion:**

TPX2 could be a prognostic biomarker for EC and play an important role in the CX3CR1/CXCL10 chemokine pathway and PI3K/Akt pathway via Sp1.

## 1 INTRODUCTION

Endometrial cancer (EC) is one of the most common malignancies of the female reproductive system and exhibits unique biological behaviors.^1^ Although some patients have typical symptoms, such as postmenopausal vaginal bleeding and vaginal discharge, which can be pathologically diagnosed via curettage, a considerable number of women regrettably lose access to treatment because of their asymptomatic state. For approximately 30% of individuals with advanced EC, overall survival (OS) does not improve even if the recommended treatment methods are adopted.^2^ To address the unsatisfactory effect of therapy and the urgent need to improve OS, the identification of prognostic factors and exploration of the corresponding molecular mechanism are important.

TPX2, a microtubule‐associated protein, is responsible for mitotic spindle assembly during mitosis, primarily by binding to, localizing with and activating Aurora A kinase.^3^, ^4^, ^5^, ^6^ Overexpression of TPX2 induces centrosome amplification and thus leads to polyploidy.^7^, ^8^, ^9^ TPX2 participates not only in chromosomal missegregation and aneuploidization but also in genomic instability, resulting in cell cycle progression in cancer cells. As observed for some mitosis‐related proteins, such as Ki‐67 and PCNA, overexpression of TPX2 is associated with poor prognosis in many cancers.^10^, ^11^, ^12^ The ERK/GSK3β/SNAIL pathway in prostate cancer,^13^ P53 pathway in breast cancer,^14^ and PI3K/AKT pathway in hepatocellular cancer^15^ have been found to be involved in TPX2‐related carcinogenic processes. However, to our knowledge, the clinical significance and molecular mechanism of TPX2 in EC are incompletely elucidated.

To reveal the biological functions and clinical relevance of TPX2 in EC, we evaluated the expression of TPX2 in the TCGA database and in‐house specimens and analyzed the relationships between the expression of TPX2 and clinicopathological parameters. We downregulated and overexpressed TPX2 in EC cell lines and observed phenotypic changes in proliferation, migration and apoptosis. Bioinformatic analyses revealed possible signaling pathways and interacting proteins related to TPX2. Coimmunoprecipitation (co‐IP) and chromatin immunoprecipitation (ChIP) were used to identify interacting proteins and promoter‐binding sequences mediating the activity of possible signaling pathways. Tumor models were established in nude mice to verify the biological functions of TPX2 in vivo. In summary, our study expanded the new research on TPX2 and revealed a mechanism by which TPX2 regulates proliferation, migration and apoptosis that could provide new ideas for the treatment of refractory EC.

## 2 MATERIALS AND METHODS

### 2.1 Bioinformatic analysis

The TCGA expression matrix (fragments per kilobase million) and clinical information were downloaded from TCGA (http://portal.gdc.cancer.gov/). The R package “limma” was employed to screen for differentially expressed genes (DEGs) with threshold criteria of |log_2_FC| >2 and adjusted *p* < 0.05. Prognostic data were downloaded from The Human Protein Atlas (HPA, https://www.proteinatlas.org/), and genes related to poor prognosis were identified. KEGG analysis, GO analysis, GSEA and prognostic analysis were carried out with the online tool XianTao (https://www.xiantaozi.com/). Intersections between sets of genes were visualized on Venn diagrams (http://bioinfogp.crib.csic.es/tools/venny).

### 2.2 Clinical specimens

A total of 609 EC tissues and 105 normal endometrial tissues were collected from the archive of formalin‐fixed, paraffin‐embedded specimens in the pathology departments of Xiangyang Central Hospital and Xiangyang First People's Hospital between February 2006 and December 2013. Relevant medical information was retrieved from the medical records database. No patient with EC underwent radiotherapy or chemotherapy prior to surgery or received hormonal therapy. The patients were followed up through the registered telephone information; for each patient, follow‐up ended on October 30, 2021, or upon his or her death. Ethical approval was granted by the medical ethics committees of Xiangyang Central Hospital.

### 2.3 Immunohistochemical (IHC) staining and evaluation

IHC staining was performed as described previously.^16^ TPX2 staining was observed in nuclei as brownish‐yellow particles. The staining intensity (SI) and the percentage of positive cells (PP) were used to calculate a semiquantitative immunoreactivity score. The SI score was assigned as follows: 0, negative; 1, weak; 2, intermediate; and 3, strong staining. The PP score was assigned as follows: 0, < 5%; 1, 5%–25%; 2, 26%–50%; 3, 51%–75%; and 4, >75% positive cell. The final score was calculated as the sum of those two scores. Four grades were determined by the final score: negative (−) expression, final score of 0 or 1; weak positive (+) expression, final score of 2 or 3; moderate positive (++) expression, final score of 4 or 5; strong positive (+++) expression, final score of >6. All sections were independently evaluated by two experienced pathologists, and if their conclusions were different, a third pathologist participated in the evaluation, and all three pathologists collaborated on the final decision.

### 2.4 Cell culture

The EC cell lines AN3CA and Ishikawa were purchased from The Cell Bank of Type Culture Collection of the Chinese Academy of Sciences and cultured in DMEM (Gibco, Carlsbad, CA, USA) supplemented with 10% FBS (HyClone, Logan, Utah, USA), 100 U/mL penicillin and 100 μg/mL streptomycin at 37°C in a humidified atmosphere of 95% O_2_ and 5% CO_2_.

### 2.5 Construction and transfection of the lentiviral shRNA vector

The lentiviral shRNA vectors were designed by, constructed by and purchased from Shanghai OBiO Technology (product number: GL427NC2‐HU6‐F2_R_D10). The three human shRNA (sh‐TPX2) sequences and the negative control (sh‐Ctrl) sequence are listed as follows:

1. sh‐TPX2#1 (Y‐17894) GAACAATCCATTCCGTCAAAT.
2. sh‐TPX2#2 (Y‐17895) CTAATCTTCAGCAAGCTATTG.
3. sh‐TPX2#3 (Y‐17896) TCCAGACCTTGCCCTACTAAG.
4. sh‐Ctrl (Y‐17894) CCTAAGGTTAAGTCGCCCTCG.

Both the sh‐TPX2 and sh‐Ctrl transfection groups were compared with the mock control group containing the transfection reagent mix without any shRNA. Approximately 2 × 10^5^ cells were seeded per well in 6‐well plates and cultured in complete culture medium to 70% confluence. Five microliters of the shRNA solution (20 μM) and 5 μL of X‐tremeGENE siRNA Transfection Reagent (Roche Diagnostics, Basel, Switzerland) were diluted separately in 100 μL of Opti‐MEM (Gibco). The diluted shRNAs and transfection reagent were then mixed together and incubated without motion for 20 min at room temperature. The transfection complex was diluted with FBS‐free DMEM to a final shRNA concentration of 80 nM and added to a six‐well plate. After incubation for 6 h, the medium was replaced with DMEM supplemented with 10% FBS. Then, 48 h post‐transfection, cells were harvested for reverse transcription–quantitative polymerase chain reaction (RT–qPCR) or Western blot analysis.

### 2.6 Construction and transfection of the TPX2 overexpression plasmid

The TPX2 overexpression plasmid (FLAG‐TPX2; product number H10559) and TPX2 empty vector (FLAG; product number H149) were designed by, constructed by and purchased from Shanghai OBiO Technology. The overexpression plasmids were transfected according to the manufacturer's protocol by a method similar to that used for transfection of the lentiviral shRNA vectors. The overexpression efficiency was evaluated by RT–qPCR and Western blotting.

### 2.7 CCK8 cell proliferation assay

A CCK8 cell proliferation detection kit was purchased from Meilunbio Biology Inc. (Dalian, China). All manipulations were performed according to the manufacturer's protocol. The calculation equations are as follows:

$$\begin{array}{c} \text{Proliferation rate}\;\left(\%\right)=\left(\text{experimental group}-\text{empty vector group}\right)/\\ \;\left(\text{control group}-\text{empty vector group}\right) \end{array}$$

$$\begin{array}{c} \text{Inhibition rate}\;\left(\%\right)=1-\left(\text{experimental group}-\text{empty vector group}\right)/\\ \;\left(\text{control group}-\text{empty vector group}\right) \end{array}$$

### 2.8 Cell cycle analysis by flow cytometry

Before cell cycle analysis, cells were subjected to cell cycle synchronization. Synchronization was performed as described previously.^17^ Cells were detached using 1 mL of trypsin (0.25%), collected in Eppendorf (EP) tubes, and centrifuged for 5 min (1500 rpm), and the supernatant was then discarded. The cells were fixed with 500 μL of 75% ethanol for 2 h and were then centrifuged, washed twice with PBS, and centrifuged again. The supernatant was then discarded. The cells were incubated with 500 μL of propidium iodide (PI)/RNaseA working solution in the dark at room temperature for 60 min. Fluorescence signals were detected and recorded at an excitation wavelength of 488 nm with a FACS system (Becton Dickinson) running Cell Quest research software (Becton Dickinson).

### 2.9 Analysis of Annexin V‐FITC/PI staining by flow cytometry

Cells were centrifuged for 5 min (1500 rpm) prior to pelleting and resuspension. The mixture was incubated with Annexin V‐FITC (Pharmingen) and PI (Pharmingen) for 15 min at room temperature in the dark prior to the addition of 400 μL of 1 × binding buffer and analysis by FACS.

### 2.10 Transwell assays

The specific procedures were performed as described previously.^18^ Images were acquired under a microscope (Olympus, Tokyo, Japan).

### 2.11 Tumor models in nude mice

AN3CA cells were stably transfected with sh‐Ctrl and sh‐TPX2. Ishikawa cells were stably transfected with FLAG and FLAG‐TPX2. Twenty nude mice (6 weeks old) were randomly divided into the FLAG‐TPX2 group, FLAG group, sh‐Ctrl group, and sh‐TPX2 group (five mice in each group). Transfected cells (8 × 10^6^) were injected subcutaneously into the right axillae of nude mice to generate tumors. The tumors were measured daily in two dimensions using a caliper. The equation volume = [1ength (mm) × width (mm)^2^]/2 was used to evaluate the tumor volume, and the nude mice were also weighed. Mice were euthanized 21 days after the injection of cells for harvesting of complete tumors.

### 2.12 Real‐time fluorescence quantitative polymerase chain reaction (RT–qPCR)

RNA extraction was performed by the TRIzol, chloroform and isopropanol methods. The thermal cycling protocol used for PCR consisted of predenaturation at 95°C for 30 s; 40 cycles of denaturation at 95°C for 15 s, annealing at 60°C for 30 s, and extension for 10 s; and a final extension step at 72°C for 1 min. GAPDH was used as a reference gene. The primers used in our study are listed in Table S1. The ΔΔCT method was employed to calculate relative expression levels.

### 2.13 Western blotting

Western blotting was carried out as described previously.^18^ GAPDH was used to normalize relative expression levels. Antibody information is listed in Table S2. We performed film scanning after development and fixation, and the optical densities of the target bands were analyzed with ImageJ software.

### 2.14 Co‐IP

Co‐IP was carried out as described previously.^19^ In brief, total protein was extracted from EC cell lines with IP lysis buffer. Protein was then quantified with a BCA Protein Assay Kit (Baiqiandu Biotech, Wuhan, China). An anti‐Sp1 antibody, anti‐TPX2 antibody (Proteintech, Cat. No: 21962‐1‐AP and 11741‐1‐AP) and protein A/G beads were incubated with the protein lysates at 4°C overnight. The immunocomplex mixture was washed with precooled IP lysis buffer five times, and immunoprecipitated complexes were then boiled in SDS–PAGE loading buffer. The subsequent loading, electrophoresis and membrane transfer procedures were similar to those used for Western blotting.

### 2.15 ChIP

An EZ‐Magna ChIP A/G Chromatin Immunoprecipitation Kit was purchased from Merck Millipore, Darmstadt, Germany. Experimental procedures were performed according to the manufacturer's instructions. In brief, cells were treated with 1% formaldehyde at room temperature for 10 min, the reaction was terminated by the addition of 10× glycine, and the suspension was then centrifuged to remove the supernatant. The cell pellets were resuspended in protease inhibitor cocktail II and were then centrifuged to collect the cells. The cells were lysed with cell lysis buffer. The cell lysates were subjected to ultrasonic fragmentation and were then incubated with the indicated antibody at 4°C overnight prior to the addition of Protein A/G magnetic beads. The beads were sequentially washed with sonication buffer and low‐salt wash buffer. The immunoprecipitated complexes were eluted from the beads and retrieved by incubation with elution buffer at 65°C for 4  h. The eluate was incubated with proteinase K and RNase A for 1 h with oscillation. Sequential centrifugation and washing with wash reagent B and elution buffer C was performed to obtain purified DNA, which was used for detection of fragments in the promoter of TPX2 by RT–qPCR. An anti‐Sp1 antibody (Proteintech, 21962‐1‐AP, dilution 1:1000) was used for this assay. The sequences of the primers specific for the TPX2 promoter were AAAAGGTGTGGTGGCTCACA (forward) and GAGACGGGGTTTCACCATGT (reverse).

### 2.16 Treatment of EC cells with the Sp1 inhibitor

The Sp1 inhibitor plicamycin (MCE, Cat. No. P113149) was used to interfere with cellular activities. Based on the instructions, 10–200 nM plicamycin was the recommended concentration. Based on the literature,^20^ we chose a median concentration of 80 nm for treatment of cells. We also treated cells with a concentration gradient of plicamycin, and the results showed that the concentration closest to the half‐maximal inhibitory concentration (IC50) was 80 nM. After the cells adhered to the wall for 24 h, the medium was discarded, and the cells were cultured with fresh medium containing Sp1 and 80 nM plicamycin. After 24 h, the cells were collected and subjected to RT–qPCR, Western blotting and other related experiments.

### 2.17 Statistical methods

SPSS 23.0 (SPSS Inc., Chicago, IL) and R software (Ver. 4.0.1) were employed for statistical analysis. Correlations between the expression level of TPX2 and clinicopathological parameters were analyzed by Student's *t* test, the chi‐squared test, and Fisher's exact test. The results of survival analyses were presented on Kaplan–Meier (KM) curves, and differences in survival were analyzed with the log‐rank test. Prognostic risk factors were identified with Cox regression models. To evaluate the specificity and sensitivity of TPX2 expression, receiver operating characteristic (ROC) analysis was utilized. Calibration analysis was used to evaluate the consistency and predictive accuracy of the prognostic model, and a modified concordance index (C‐index) was established to determine the discriminatory power and predictive accuracy of the nomogram. All the assays mentioned above were carried out three times, and all the results are presented as the mean ± standard deviation (SD) values. A two‐sided *p* of <0.05 was considered to indicate a statistically significant difference.

## 3 RESULTS

### 3.1 TCGA data analysis revealed that TPX2 could be an independent prognostic factor

The clinical characteristics of the patients are listed in Table S3. A total of 1593 DEGs were identified, of which 645 were upregulated and 948 were downregulated (Figure S1A). The top 50 genes with markedly upregulated expression were used for subsequent analysis (Figure S1B). The genes associated with poor prognosis in EC were searched in and downloaded from The Human Protein Atlas, and the top 250 genes with the smallest *p* values were retained. The intersection between these two gene sets was visualized on a Venn diagram. Two genes, NCAPG and TPX2, were identified by this screen (Figure S1C). The relationship between TPX2 and poor prognosis has been established in several malignancies; however, the clinical significance and possible molecular mechanism of TPX2 in EC have rarely been reported. This knowledge gap regarding TPX2 in EC prompted us to investigate further.

A total of 552 patients represented in the TCGA dataset with complete clinical information, including histological grade, clinical stage, pathological type and survival time., were included in our study. The detailed clinical information is listed in Table S3. The expression of TPX2 in cancer tissues was significantly higher than that in adjacent normal tissues (Figure S2A).

As shown in Figure S6B, the expression of TPX2 was significantly correlated with age (*p* < 0.001), clinical stage (*p* < 0.001), histological type (*p* < 0.001), and histological grade (*p* < 0.001). High expression of TPX2 conferred poor prognosis, according to the results of KM survival analysis (*p* = 0.003, Figure S2C). In the subgroup analysis, age ≥ 60 years (*p* = 0.002), clinical stage III and IV disease (*p* = 0.034), BMI >30 (*p* = 0.023), absence of radiotherapy (*p* < 0.001) and menopause (*p* < 0.001) were the risk factors associated with higher expression of TPX2 that were significantly associated with poorer prognosis (Figure S2C).

ROC analysis showed that the area under the ROC curve (AUC) was 0.976, suggesting that TPX2 has superior diagnostic value (Figure S2D). Subgroup analysis showed that the AUC value for histological grade 2 (G2) versus G3 was 0.783, for G3 versus G1&G2 was 0.821, for stage III versus stage IV was 0.528, for stage I versus stage II was 0.619, and for serous cancer versus endometrioid cancer was 0.819, suggesting that TPX2 has considerable diagnostic value for histological type and histological grade (Figure S2D).

Univariate and multivariate Cox regression analyses showed that TPX2 was an independent risk factor correlated with poor prognosis (HR = 1.281, 95% CI = 1.098–1.534, *p* = 0.005). Moreover, an age >60 years, a high TP53 expression level, complete remission status, mixed histological type, treatment with radiation therapy and advanced histological grade (G2 or G3) were also identified as independent risk factors for poor prognosis in EC (Table S4).

### 3.2 Relationships between TPX2 expression and clinicopathological parameters in in‐house specimens

**FIGURE 1:**
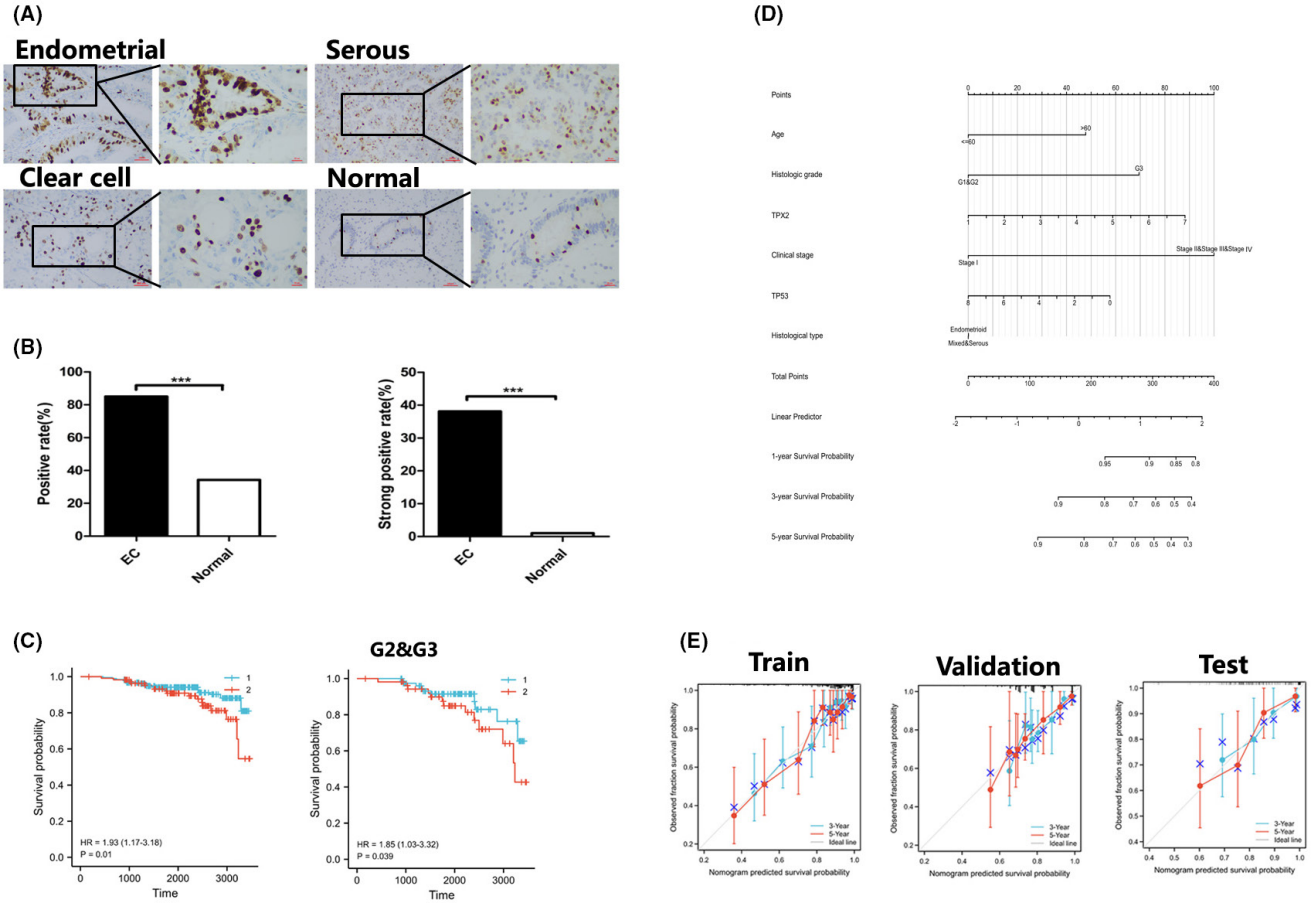
TPX2 expression and validation of the prognostic model with in‐house specimens. (A) IHC images showing TPX2 expression in different EC histological types and normal endometrial tissues. (B) Significant differences in the rates of positive and strong positive TPX2 expression were found between tumor and normal tissues. (C) OS and subgroup (G2 & G3) analysis results presented on KM survival curves. (D) Nomogram for predicting OS probabilities at 1, 3 and 5 years. (E) The calibration model for predicting OS probabilities at 3 and 5 years was established in the training cohort and verified in the validation cohort. Calibration analysis curves for clinical prediction of 3‐year or 5‐year survival are shown from the test cohort.

The clinicopathological parameters of 609 individuals are listed in Table S5. The rates of positive and strong positive TPX2 expression were significantly higher in cancer tissues, including those of endometrioid cancer, serous cancer and clear cell cancer, than in the corresponding normal endometrial tissues (*p* < 0.001, Figure 1A,B).

The relationships between these two TPX2 expression states (positive and strong positive) and clinicopathologic parameters, including EC type, pathological type, clinical stage, histological grade, tumor invasion status, P53 status, Ki67 proliferation index, and lymph node metastasis status, were analyzed with the chi‐square test (Table S6). For clinical stages, the rates of both positive and strong positive TPX2 expression showed significant differences (*p* = 0.001 and *p* < 0.001, respectively). For tumor invasion status and lymph node metastasis status, a significant difference existed in the rate of strong positive TPX2 expression (both *p* < 0.001). The rate of positive TPX2 expression showed significant differences for EC type and histological type (*p* = 0.003 and *p* < 0.001, respectively).

High expression of TPX2 was associated with poor prognosis according to the results of KM survival analysis (Figure 1C). In the subgroup analysis, G2 and G3 conferred poorer prognosis (*p* < 0.05). Cox univariate regression analysis showed that TPX2 expression level, histological type, patient age, clinical stage, histological grade, myometrial invasion status, lymph node metastasis status and Ki67 index were significantly correlated with OS (*p* < 0.05, Table S7). Cox multivariate regression analysis revealed that deep myometrial invasion, advanced clinical stage, and lymph node metastasis, but not TPX2 expression, were independent risk factors for EC (*p* < 0.05, Table S7).

### 3.3 Establishment and validation of a prognostic model of EC

The patients represented in TCGA were grouped randomly into a training cohort (70% of the patients, *n* = 380) and a validation cohort (30%, *n* = 162). Independent risk factors identified by multivariate Cox regression analysis were included in analysis of the training set, and a prognostic nomogram was constructed to predict the 1‐, 3‐, and 5‐year survival probabilities (Figure 1D). The calibration curve was constructed and showed that the C‐indices in the training cohort and the validation cohort were 0.791 (95% CI: 0.763–0.819) and 0.730 (95% CI: 0.684–0.775), respectively, and the curves were at an angle of approximately 45 degrees, showing that the model had good discrimination ability and correction performance (Figure 1E). The clinical efficacy of the prognostic model established from TCGA data was tested with in‐house specimens. The calibration curve was constructed and showed that the C‐index of the model in the test cohort was 0.813 (95% CI: 0.787–0.839), and the curve was at an angle of approximately 45 degrees, indicating that the model had good discrimination ability and predictive accuracy (Figure 1E).

### 3.4 TPX2 regulated the phenotypes of AN3CA and Ishikawa cells

**FIGURE 2:**
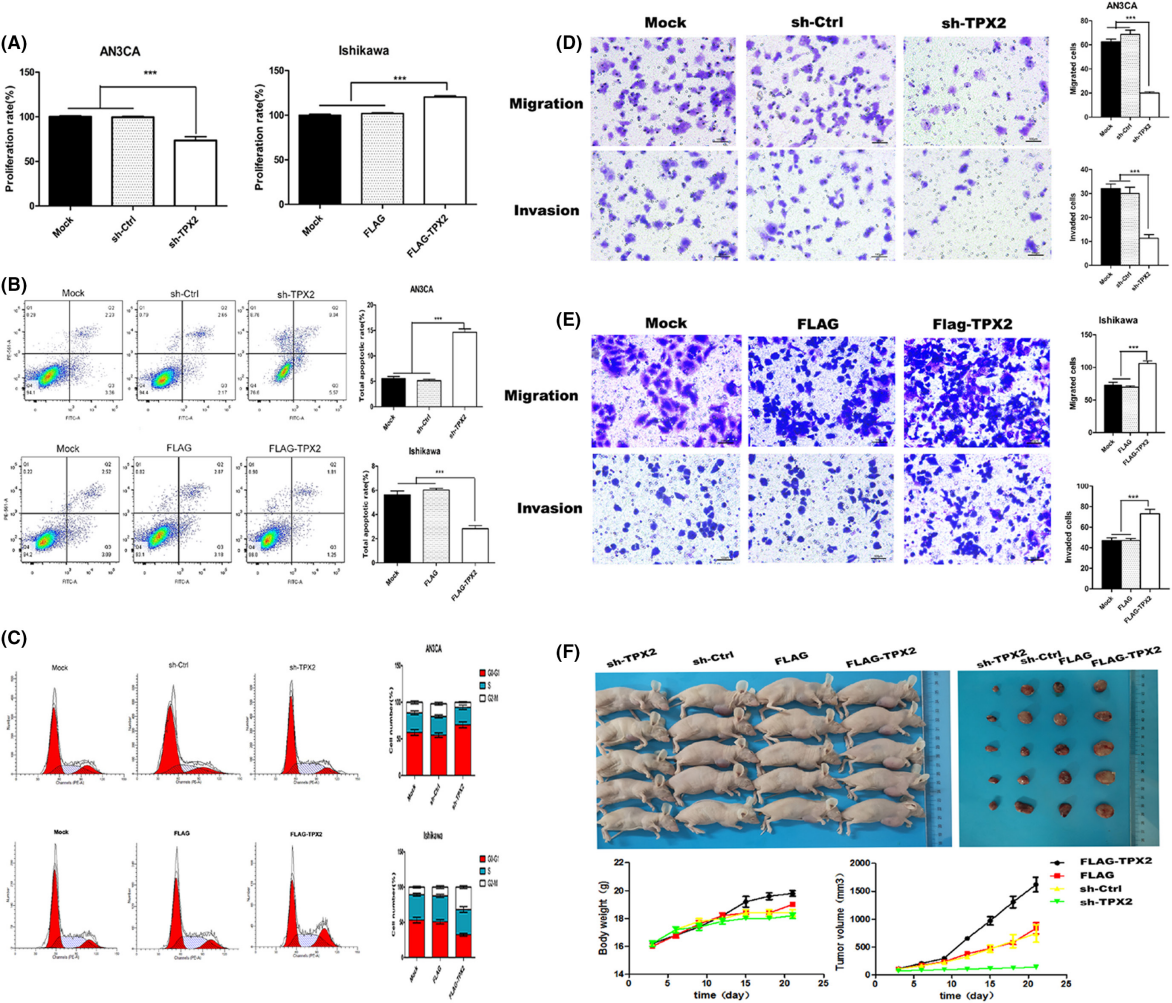
TPX2 regulated the proliferation, migration and invasion of AN3CA and Ishikawa cells. (A) Effects of TPX2 knockdown and overexpression on the proliferation of AN3CA and Ishikawa cells in CCK8 assays. (B) Changes in apoptosis in AN3CA and Ishikawa cells induced by TPX2 knockdown and overexpression were detected with flow cytometry. (C) Effects of TPX2 knockdown and overexpression on cell cycle changes in AN3CA and Ishikawa cells. Changes in the migration and invasion of cells induced by TPX2 knockdown (D) and TPX2 overexpression (E). (F) Effects of TPX2 knockdown and overexpression on the growth of transplanted tumors in a nude mouse model.

The CCK8 cell proliferation assay showed that the proliferation of the AN3CA cell line was inhibited by TPX2 knockdown (*p* < 0.001) and the proliferation of the Ishikawa cell line was enhanced by TPX2 overexpression (*p* < 0.001, Figure 2A).

Compared with that in the mock and sh‐Ctrl groups, the apoptosis rate of AN3CA cells was significantly increased with TPX2 knockdown (*p* < 0.001), and compared with that in the mock and Flag groups, the apoptosis rate of Ishikawa cells was significantly decreased with TPX2 overexpression (*p* < 0.001, Figure 2B).

The number of TPX‐2‐overexpressing Ishikawa cells in G2/M phase was increased significantly compared with those in the mock and FLAG groups (*p* < 0.001), and the number of AN3CA cells with TPX2 knockdown in G0‐G1 phase was increased significantly compared with those in the mock and sh‐Ctrl groups (*p* < 0.001, Figure 2C).

With knockdown of TPX‐2 expression, the migration and invasion abilities of AN3CA cells were significantly attenuated compared with those of the mock and sh‐Ctrl cells (*p* < 0.001, Figure 2D). With overexpression of TPX‐2, the migration and invasion abilities of Ishikawa cells were enhanced compared with those of the mock and FLAG cells (*p* < 0.001, Figure 2E).

In the tumor model in nude mice, the growth of transplanted tumors in the sh‐TPX2 group was significantly inhibited compared with that in the sh‐Ctrl group. In addition, the transplanted tumors were smaller and the weights of the nude mice were lower in the sh‐TPX2 group than in the sh‐Ctrl group (*p* < 0.01). Conversely, the growth of transplanted tumors was enhanced, the size of the transplanted tumors was increased, and the weight of the nude mice was increased in the FLAG‐TPX2 group compared with the FLAG group (*p* < 0.01, Figure 2F).

### 3.5 TPX2 regulated the CX3CR1/CXCL10 chemokine pathway

**FIGURE 3:**
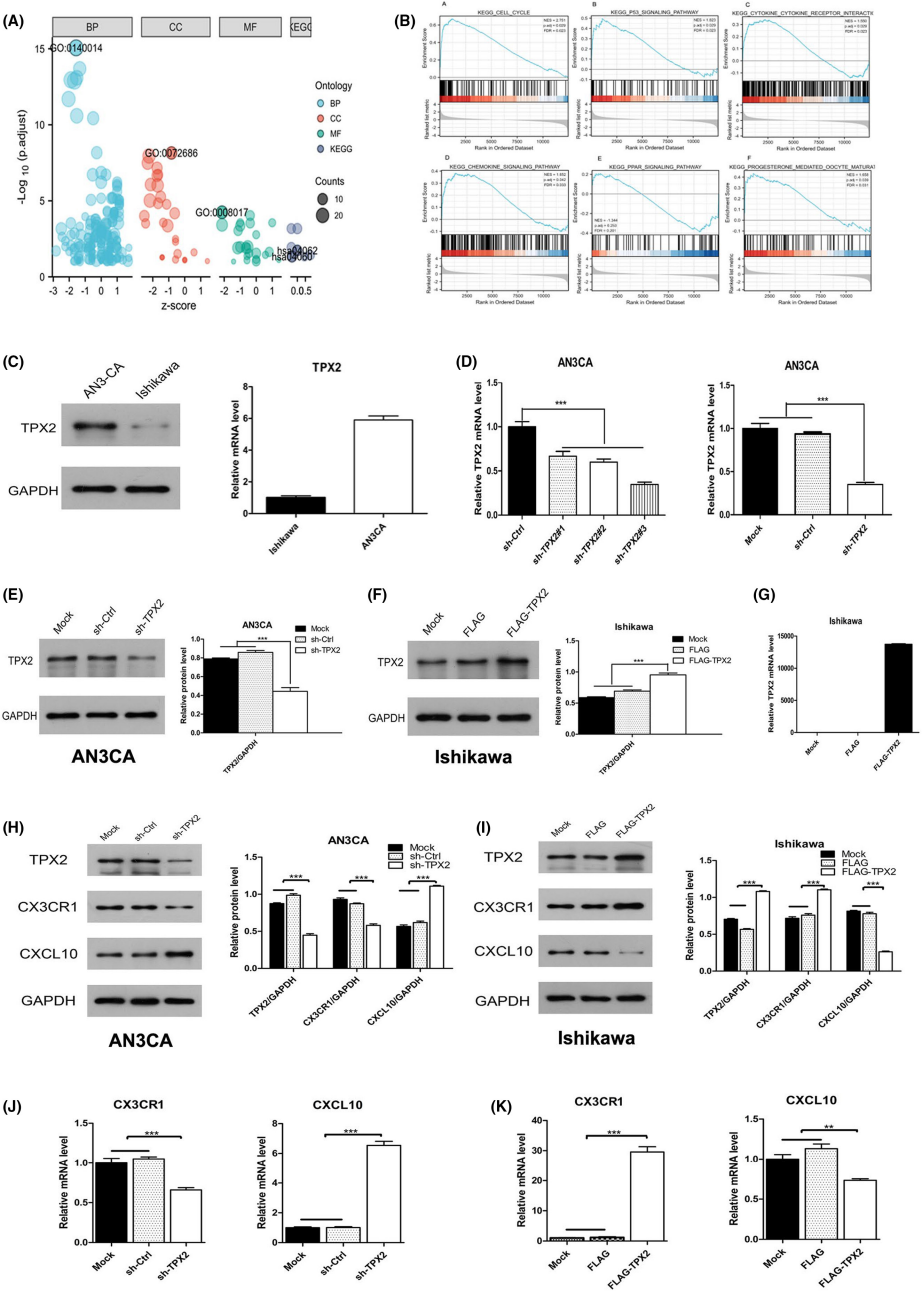
TPX2 regulated the CX3CR1/CXCL10 chemokine pathway. (A) Results of GO and KEGG analyses based on DEGs in cells with TPX2 overexpression, presented on a bubble diagram. The top 5 terms/pathways with the lowest p values are annotated. (B) Enrichment plots from GSEA. (C) The protein expression levels of TPX2 presented by Western blotting in AN3CA and Ishikawa cells. (D) The most effective interference sequence (sh‐TPX#3) was chosen from the three sh‐TPX2 interference sequences and the mRNA expression levels of TPX2 were compared by RT–qPCR in AN3CA cells transfected with sh‐TPX2 and sh‐Ctrl. (E) the protein expression levels of TPX2 were detected by Western blotting in AN3CA cells transfected with sh‐TPX2 and sh‐Ctrl. The protein (F) and mRNA (G) expression levels of TPX2 were presented by Western blotting in Ishikawa cells transfected with FLAG‐TPX2 or FLAG. (H) The protein expression levels of TPX2, CX3CR1 and CXCL10 were measured by Western blotting in AN3CA cells transfected with either sh‐TPX 2 or sh‐Ctrl. (I) The protein expression levels of TPX2, CX3CR1 and CXCL10 were measured by Western blotting in Ishikawa cells transfected with either FLAG‐TPX2 or FLAG. (J) The mRNA expression levels of CX3CR1 and CXCL10 were compared by RT–qPCR in AN3CA cells transfected with sh‐TPX2 and sh‐Ctrl. (K) The mRNA expression levels of CX3CR1 and CXCL10 were compared by RT–qPCR in Ishikawa cells transfected with either FLAG‐TPX2 or FLAG.

Gene ontology (GO) term analysis showed enrichment in multiple functions, and the characteristic functions with the lowest *p* values are listed as follows: in the biological process (BP) category, GO: 0140014—mitotic nuclear division (*p* < 0.001); in the cellular component (CC) category, GO: 0072686—mitotic spindle (*p* < 0.001); and in the molecular function (MF) category, GO: 0008017—microtubule binding (*p* < 0.001, Figure 3A). Kyoto Encyclopedia of Genes and Genomes (KEGG) pathway analysis showed enrichment in eight pathways: cell cycle, PPAR signaling pathway, progesterone‐mediated oocyte maturation, human *T*‐cell leukemia virus 1 infection, p53 signaling pathway, chemokine signaling pathway, cytokine–cytokine receptor interaction, and IL‐17 signaling pathway. Among these pathways, chemokine signaling pathway (hsa04062) and cytokine–cytokine receptor interaction pathway (hsa04060) had not been investigated previously and attracted our interest (Figure 3A). Gene set enrichment analysis (GSEA) showed that the cell cycle, PPAR signaling pathway, progesterone‐mediated oocyte maturation, human *T*‐cell leukemia virus 1 infection, p53 signaling pathway, chemokine signaling pathway, cytokine–cytokine receptor interaction, and IL‐17 signaling pathway were the main enriched pathways (Figure 3B). The chemokine signaling pathway overlapped between the KEGG analysis and GSEA results. Therefore, we selected this pathway for further verification and selected the representative CX3CR1 and CXCL10 genes in the chemokine pathway for verification of expression. We measured the basal expression level of TPX2 in AN3CA and Ishikawa cells and found that it was relatively low in Ishikawa cells and relatively high in AN3CA cells. Therefore, we chose Ishikawa cells as the experimental cells for upregulating TPX2 expression and AN3CA cells as the experimental cells for downregulating TPX2 expression (Figure 3C–G). Compared with those in the mock and sh‐Ctrl cells, the CX3CR1 protein and mRNA expression levels in TPX2‐knockdown AN3CA cells were significantly decreased, while the CXCL10 protein and mRNA expression levels were significantly increased (all *p* < 0.001). In Ishikawa cells with TPX2 overexpression, the CX3CR1 protein and mRNA levels were significantly increased but the CXCL10 protein and mRNA expression levels were significantly decreased compared with those in the mock and FLAG cells (*p* < 0.001 for CX3CR1, *p* < 0.01 for CXCL10; Figure 3H–K).

### 3.6 TPX2 activated the PI3K/Akt pathway

**FIGURE 4:**
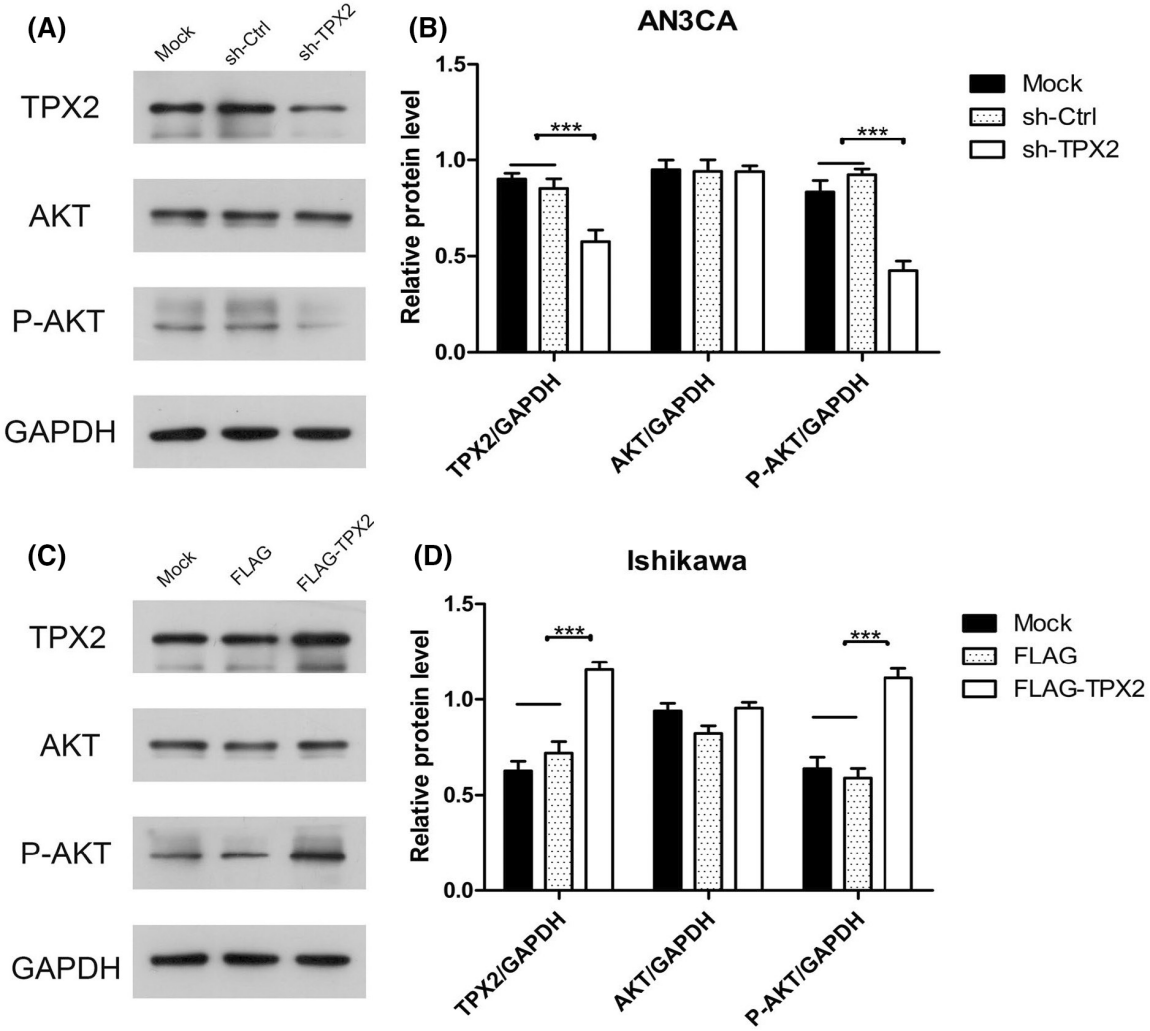
TPX2 regulates the PI3K/Akt pathway. (A) The level of phosphorylated Akt in AN3CA cells with TPX2 knockdown was measured by Western blotting. (B) The relative ratio of the detected protein to GAPDH was presented as a histogram. (C) The level of phosphorylated Akt in Ishikawa cells with TPX2 overexpression was measured by Western blotting. (D) A hitogram reflected the relative ratio of the phosphorylated Akt to GAPDH.

Through the KEGG website (https://www.kegg.jp/), we found that the chemokine pathway was coupled to the PI3K‐Akt pathway, and the downstream effects of chemokine pathway signaling could directly activate the PI3K‐Akt pathway (Figure S3). We explored the relationship between TPX2 and the PI3K/Akt pathway in EC cells with upregulated/downregulated TPX2 expression by Western blot analysis. Compared with the mock and sh‐Ctrl groups, the protein level of p‐Akt was reduced in AN3CA cells with TPX2 knockdown, although the level of total Akt protein was barely affected (*p* < 0.001, Figure 4A,B). In Ishikawa cells with TPX2 overexpression, the protein level of p‐Akt was higher than that in mock or FLAG cells, and the level of total Akt protein was barely affected (*p* < 0.001, Figure 4C,D).

### 3.7 Sp1 interacted with TPX2 and regulated its expression

**FIGURE 5:**
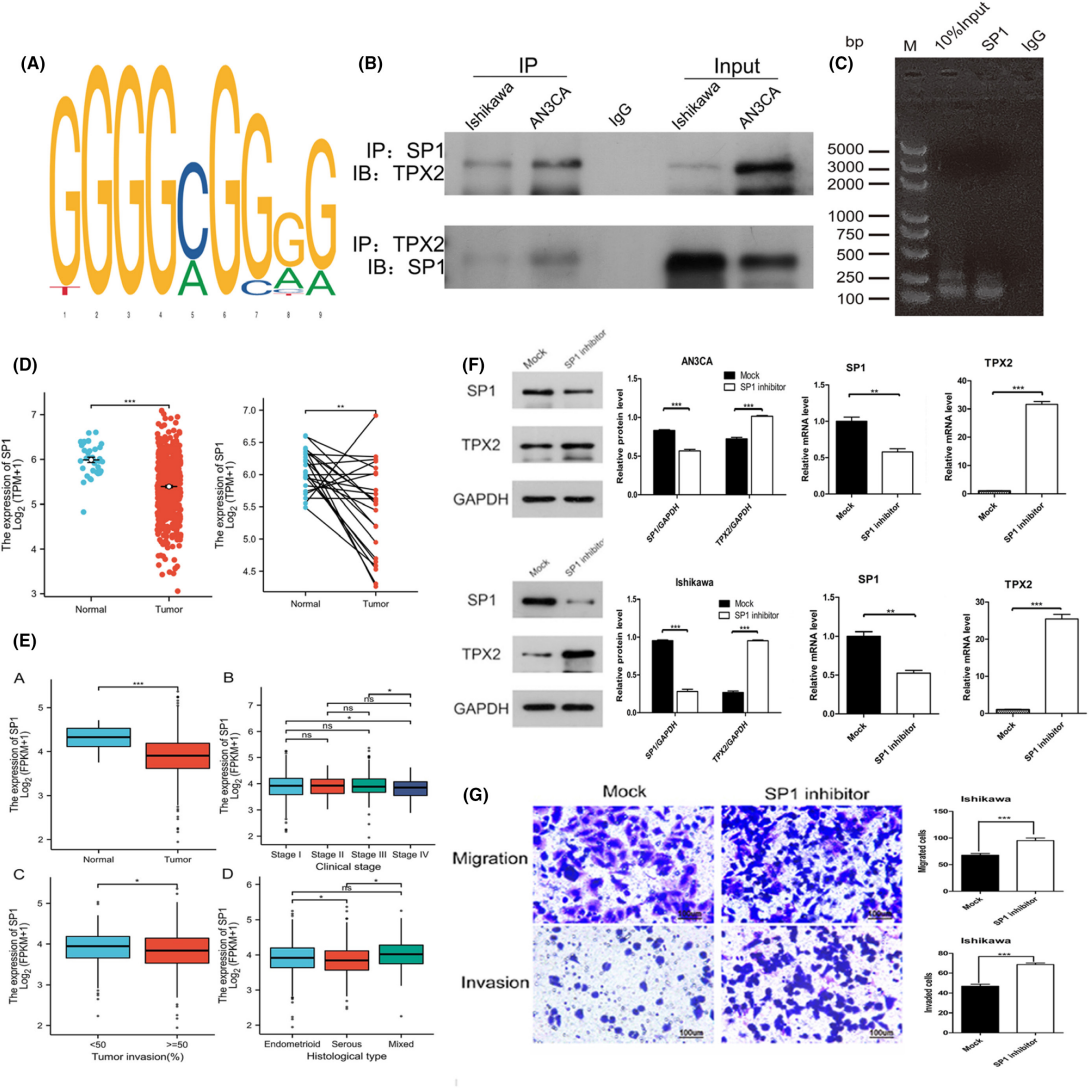
Sp1 interacted with TPX2 and regulated its expression by binding to its promoter. (A) The potential Sp1 binding site in the TPX2 promoter was predicted with JASPAR. (B) The protein–protein interaction between TPX2 and Sp1 was detected by immunoprecipitation in AN3CA and Ishikawa cells. (C) The binding of Sp1 to the TPX2 promoter region was determined by ChIP and RT–qPCR in Ishikawa cells. (D) Significant differences in SP1 expression between tumor and normal tissues in the paired and unpaired groups are shown on dot plots. (E) The relationships between Sp1 expression and clinical parameters are shown on box‐and‐whisker plots. (F) The expression levels of Sp1 and TPX2 in AN3CA and Ishikawa cell lines treated with the Sp1 inhibitor were measured by Western blotting and are shown in histograms. (G) Changes in the migration and invasion of Ishikawa cells after treatment with the Sp1 inhibitor.

The transcription factors of TPX2 were predicted with JASPAR (http://jaspar.genereg.net/), and those with the highest scores were selected (Figure 5A). We also analyzed the relationships between the expression of SP1 and the clinicopathological parameters of patients with EC in the TCGA database. The mRNA level of Sp1 in EC tissues was decreased compared with that in normal tissues (*p* < 0.001, Figure 5D), and low expression of Sp1 was significantly correlated with a clinical stage of IV, a tumor invasion depth of ≥50%, and specific histological types such as endometrioid and mixed (all *p* < 0.05, Figure 5E).

To further analyze the relationship between Sp1 and TPX2, immunoprecipitation and ChIP–PCR experiments were used to verify the direct binding of Sp1 to TPX2 (Figure 5B,C). In the immunoprecipitation experiment, the direct protein–protein interaction (Figure 5B) between Sp1 and TPX2 in AN3CA and Ishikawa cells was verified. In Ishikawa cells, the background expression of the TPX2 protein was low, while Sp1 was strongly expressed. On the other hand, in AN3CA cells, the background expression of the TPX2 protein was high, while that of Sp1 was low, further indicating the negative regulation of TPX2 by Sp1 (Figure 5B).

We performed a ChIP assay on Ishikawa cells to detect the interaction between Sp1 and TPX2 and found that Sp1 could directly bind to the TPX2 promoter region. Thus, it was confirmed that Sp1 is a transcription factor of TPX2 and that Sp1 can interact with TPX2 in AN3CA and Ishikawa cells. (Figure 5C).

We found through a literature search and preliminary experiments that for the Sp1 inhibitor plicamycin, a concentration of 80 nM was the approximate the IC50 against SP1 (Figure S4A,B). Thus, plicamycin (80 nM) was added to the culture medium of AN3CA and Ishikawa cells. Compared with the mock treatment, plicamycin suppressed the expression of the Sp1 protein but enhanced the expression of the TPX2 protein (*p* < 0.001); moreover, it considerably reduced the expression of Sp1 mRNA but significantly increased that of TPX2 mRNA (*p* < 0.001). These results indicated that Sp1 could target TPX2 and regulate its expression. In Ishikawa cells, the expression levels of TPX2 protein and mRNA were increased significantly after addition of the Sp1 inhibitor (Figure 5F). The results of transwell assays showed that treatment with the Sp1 inhibitor significantly enhanced the migration and invasion abilities of Ishikawa cells in vitro (Figure 5G).

### 3.8 Sp1 mediated the coupling of the CX3CR1/CXCL10 chemokine pathway to the PI3K/Akt pathway through targeted inhibition of TPX2

**FIGURE 6:**
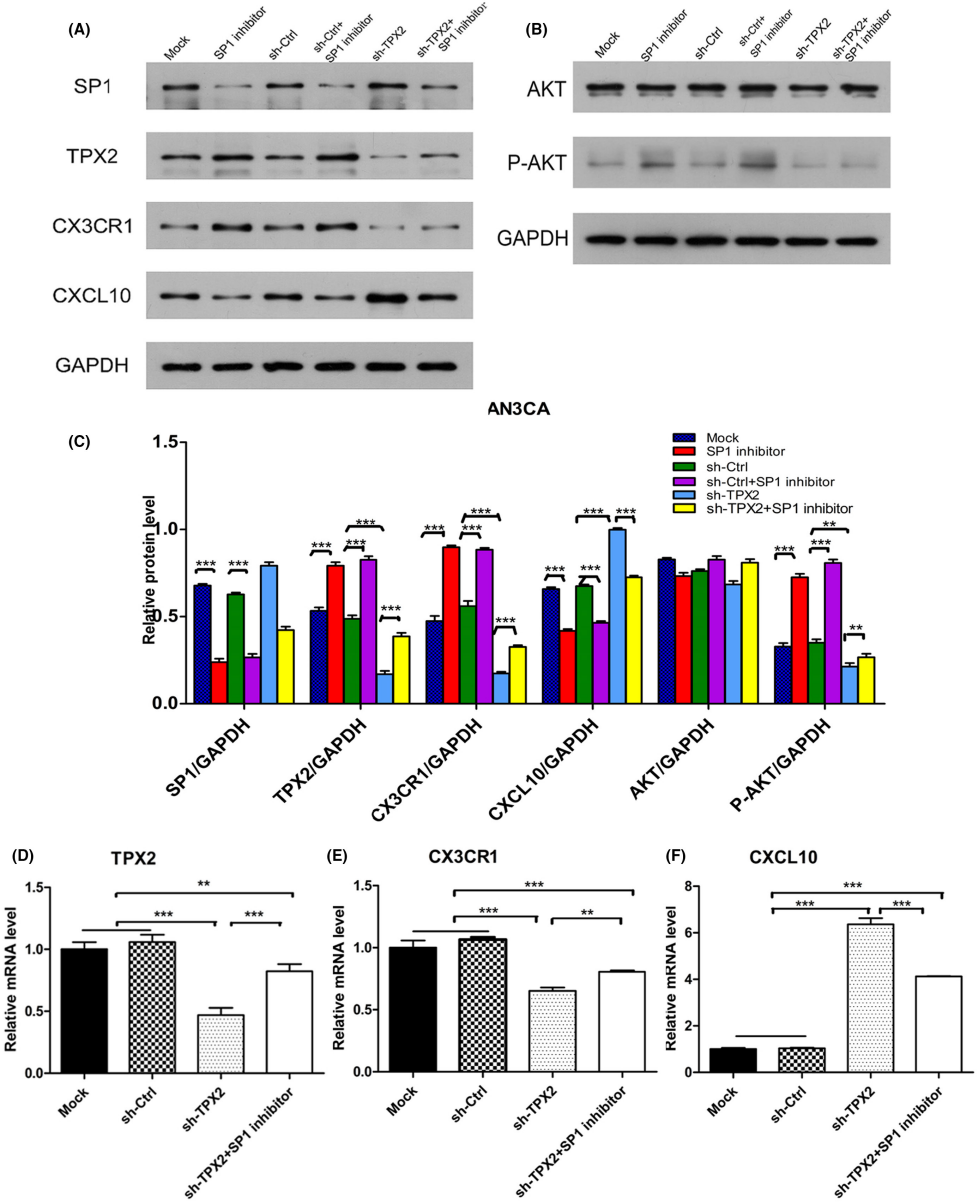
Rescue experiments in AN3CA cells. (A, B) Sp1 inhibitor, sh‐Ctrl, Sp1 inhibitor +sh‐Ctrl, sh‐TPX2, and Sp1 inhibitor +sh‐TPX2 were transfected separately into AN3CA cells, and the protein levels of related molecules such as Sp1, TPX2, CX3CR1, CXCL10, Akt, and p‐Akt were measured by Western blotting. Quantitative results are presented in a histogram (C). (D, E, F) Mock, sh‐Ctrl, sh‐TPX2, and Sp1 inhibitor +sh‐TPX2 were transfected into AN3CA cells, and the mRNA expression levels of related molecules such as Sp1, TPX2, CX3CR1, and CXCL10 were measured by RT–qPCR.

To verify whether the regulation of the CX3CR1/CXCL10 chemokine pathway and PI3K/Akt pathway by TPX2 is mediated through Sp1, rescue experiments were conducted. In AN3CA cells, the Sp1 inhibitor, sh‐Ctrl, Sp1 inhibitor +sh‐Ctrl, sh‐TPX2, and Sp1 inhibitor +sh‐TPX2 were separately transfected into AN3CA cells, and mock‐transfected cells were used as the control group. The Western blot bands are shown in Figure 6A,B. The mRNA and protein levels of TPX2 were found to decrease with knockdown of TPX2. Inhibiting the expression of Sp1 significantly reversed the downregulation of TPX2 mRNA and protein expression (*p* < 0.01). With knockdown of TPX2, the mRNA and protein levels of CX3CR1 decreased. Inhibiting the expression of Sp1 significantly reversed the downregulation of CX3CR1 mRNA and protein expression (*p* < 0.01). Similarly, the expression of CXCL10 mRNA and protein increased with knockdown of TPX2, while plicamycin treatment significantly reversed the upregulation of CXCL10 mRNA and protein expression (*p* < 0.001). Regarding the protein levels of Akt and p‐Akt, compared with mock treatment, plicamycin treatment increased the level of the p‐Akt protein (*p* < 0.001). Compared with that in the mock group, the level of p‐Akt decreased with knockdown of TPX2; moreover, inhibiting the expression of Sp1 significantly reversed the decrease in the p‐Akt level (*p* < 0.001), although the level of Akt did not change significantly. All the differences in expression are presented on histograms (Figure 6C–F).

In addition, we transfected the Sp1 inhibitor, FLAG, FLAG + Sp1 inhibitor, FLAG‐TPX2, and FLAG‐TPX2 + Sp1 inhibitor separately into Ishikawa cells and analyzed the mRNA and protein expression of downstream molecules by Western blotting and RT–qPCR. Both the mRNA and protein levels of TPX2 increased with FLAG‐TPX2 transfection and increased further with combined interference with Sp1 (*p* < 0.05, Figure S5A,C,D). FLAG‐TPX2 transfection promoted increases in the mRNA and protein level of CX3CR1, which continued to increase with combined interference with Sp1 (*p* < 0.01, Figure S5A,C,E). After FLAG‐TPX2 transfection, the CXCL10 mRNA and protein levels were decreased and were decreased further with combined interference with Sp1 (*p* < 0.001, Figure S5A,C,F). In addition, the level of the p‐Akt protein increased with FLAG‐TPX2 transfection and increased further with the combined interference with Sp1 (*p* < 0.05, Figure S5B,C). However, the Akt expression level did not change significantly (Figure S5B,C).

## 4 DISCUSSION

TPX2 is a microtubule‐associated protein located on human chromosome 20q11.2 that is responsible for cell cycle control and plays a key role in mitosis and spindle assembly. Increased expression of TPX2 has been found in some malignancies, such as esophageal cancer, colon cancer and breast cancer.^4^, ^5^, ^6^, ^14^ In this study, compared with that in normal tissues, the expression level of TPX2 was significantly higher in both TCGA data and in‐house specimens. However, the role of TPX2 in endometrial carcinogenesis is not clear. Significant associations were found between TPX2 expression and clinicopathological parameters. KM survival analysis of TCGA data showed that higher TPX2 expression conferred a shorter OS time. ROC analysis revealed the high diagnostic value of TPX2 in EC, and multivariate Cox regression analysis further indicated that high TPX2 expression is an independent risk factor in EC patients. A significant correlation was identified between positive or strongly positive TPX2 expression and higher clinical stage.

EC can be divided into four different molecular subgroups,^21^ based on the results of high‐throughput sequencing. However, many patients cannot afford high‐throughput sequencing due to its cost. Therefore, gene expression profiles have been used to identify biomarkers of EC and to establish prognostic models. According to the literature, these models are regrettably limited to a specific stage or grade. Yang et al. used a reversed‐phase protein array to construct a prognostic model for patients with early EC.^22^ Moreover, immune, metabolic or autophagy‐related coding genes and noncoding lncRNAs have been recognized as prognostic factors for EC.^23^, ^24^, ^25^, ^26^ However, prognostic models based on gene expression alone do not consider clinical and pathological characteristics, imposing limitations. It is thus necessary to establish a prognostic model that combines clinicopathological characteristics with molecular phenotypes and specific biomarkers. In this study, multivariate Cox regression analysis with TCGA data confirmed that high expression of TPX2, an age of >60 years, high expression of TP53, the serous subtype, advanced clinical stage, and a histological grade of G2 or G3 are independent risk factors for the prognosis of EC. Univariate Cox regression analysis yielded results similar to those for the TCGA data with in‐house specimens, but TPX2 was not revealed as an independent prognostic factor by multivariate Cox regression analysis, possibly due to the limited sample size. The prognostic model established based on these risk factors exhibited good validation efficiency and high consistency.

Activation of the PI3K/Akt/mTOR signaling pathway increases cell viability and enhances cell migration through upregulation of TPX2 expression and inhibits EC cell apoptosis. In liver, ovarian, breast and other cancers, overexpression of TPX2 can activate the PI3K/Akt pathway.^14^, ^15^, ^27^ In our study, TPX2 overexpression promoted the proliferation of AN3CA and Ishikawa cells, reduced apoptosis in these cells, and promoted G2/M progression in these cells, as determined by a CCK8 proliferation assay and flow cytometric analysis. In addition, loss of TPX2 inhibited cell migration and invasion. In the nude mouse tumorigenesis assay, TPX2 knockdown significantly inhibited the growth of transplanted tumors. These results revealed that TPX2 plays a vital role in the endometrial carcinogenesis, and the observed phenotypes were consistent with the results of our statistical analysis of TCGA data and in‐house specimens.

GO analysis revealed that TPX2 functions primarily in mitotic nuclear division, mitotic spindle formation, and microtubule binding, consistent with previous reports.^9^ Through integrated KEGG analysis and GSEA, several pathways were found to be closely correlated with high TPX2 expression as well as chemokine expression. The relationships between chemokines and TPX2 were analyzed. Chemokines are a class of low‐molecular‐weight cytokines secreted by cells. Cytokines usually function as signaling proteins secreted by immune cells, but tumor cells also secrete large amounts of chemokines.^28^, ^29^ In the tumor microenvironment, the secretion of chemokines induces the chemotaxis of surrounding target cells.^30^, ^31^ The identification of this signaling pathway highlights various types of chemokines and their receptors, as well as the mechanism by which chemokine signaling activates the JAK/STAT, Ras, ERK and Akt pathways and interactions of signaling pathway proteins such as CX3CR1 and CXCL10 to regulate tumor cell proliferation, migration, invasion and cell cycle progression.

We measured the expression of CX3CR1 and CXCL10, the main signaling pathway proteins, in EC cells with downregulated or upregulated TPX2. TPX2 knockdown significantly reduced the protein expression of CX3CR1 and significantly increased the protein expression of CXCL10, while TPX2 overexpression significantly increased the protein expression of CX3CR1 and significantly reduced the protein expression of CXCL10. chemokines have become new targets for tumor therapy.^32^, ^33^, ^34^ However, the regulatory effects of chemokines on tumor biological behavior are variable and are closely related to tumor type, the expression of chemokines and the tumor microenvironment. CX3CR1 is expressed in various types of cancer cells,^35^, ^36^, ^37^ transmitting signals induced by CX3CL1 and participating in the proliferation and metastasis of malignant tumors.^38^, ^39^ Ian H et al. reported that loss of CX3CR1 in the myeloid compartment in the central nervous system (CNS) triggered the malignant cycle mediated by CXCL10 and educated the microenvironment of brain metastasis (Br‐Met) to promote immunosuppression, and a negative direct correlation between CX3CR1 and CXCL10 expression was revealed.^40^ CTHRC1 not only interacts with the integrin β3‐Akt signaling pathway to increase myometrial invasion in the tumor microenvironment of EC but also promotes M2‐like tumor‐associated macrophage (TAM) invasion by increasing the expression of CX3CR1 in macrophages.^41^

Through the KEGG website (https://www.kegg.jp/), we found that the chemokine pathway can directly activate the PI3K‐Akt pathway. The expression of TPX2 is related to the phosphorylation of Akt in a variety of malignant tumors, such as liver,^15^ ovarian,^27^ and breast cancers.^14^ We speculated that TPX2 may perform a similar function in Akt phosphorylation in EC. Our study showed that compared with the mock and sh‐Ctrl groups, the level of P‐Akt in TPX2 knockdown AN3CA cells was significantly decreased. In Ishikawa cells with TPX2 overexpression, compared with the mock group and FLAG group, the P‐Akt protein level was significantly increased, but the total Akt protein level did not differ significantly, indicating that TPX2 can activate the PI3K/Akt pathway.

Subsequently, we confirmed that Sp1 can directly bind to the TPX2 promoter region and cause transcriptional inhibition of TPX2 in EC cells. TPX2 is the direct transcriptional target of Sp1. Therefore, we speculate that Sp1 specifically inhibits the expression of TPX2 at the transcriptional level. Through subsequent experiments, we found that Sp1 inhibitor treatment increased the mRNA and protein expression levels of TPX2, consistent with our speculation that TPX2 expression is inhibited via Sp1 transcription. In AN3CA EC cells, the rescue experiment also showed that Sp1 inhibitor treatment reversed the decrease in TPX2 expression caused by TPX2 knockdown. Moreover, in AN3CA cells, the protein levels of TPX2, CX3CR1 and P‐Akt, which were decreased due to TPX2 knockdown, were significantly restored and the increase in the level of CXCL10 due to TPX2 knockdown was significantly reversed after intervention with the Sp1 inhibitor.

Sp1 is a low‐molecular‐weight nuclear transcription factor that belongs to the Sp/Kruppel‐like factor (KLF) transcription factor family and contains 785 amino acids. Sp1 regulates the expression of many genes and plays a key role in a variety of biological processes.^42^ Sp1 can act on both oncogenes and tumor suppressor genes and is deeply involved in the onset and development of malignant tumors. Proto‐oncogenes such as c‐myc, ras and N‐myc and tumor suppressor genes such as p53, pRB, and p21 have been reported to be transcriptionally regulated by Sp1 in different cancers.^43^, ^44^ We observed that Sp1 regulates the oncogene TPX2 and specifically inhibits its expression in EC. Inhibiting Sp1 expression could stimulate the malignant progression of EC by upregulating the expression of TPX2, mediating the coupling of the CX3CR1/CXCL10 chemokine pathway to the PI3K/Akt pathway.

In conclusion, this research showed for the first time that the TPX2 gene is considerably upregulated in EC and that overexpression of TPX2 is closely correlated with clinicopathological parameters. This is the first study to prove that TPX2 is correlated with poor prognosis in individuals with EC. Sp1 regulates TPX2 expression, resulting in a cascade effect, in EC; it affects the malignant progression of endometrial cancer cells by coupling the CX3CR1/CXCL10 chemokine pathway to the PI3K/Akt signaling pathway. TPX2 and Sp1 were identified in our study as molecules closely correlated to the phenotype of EC cells, providing promising therapeutic targets for precision treatment of EC.

## AUTHOR CONTRIBUTIONS

**Mei Yang:** Data curation (equal); formal analysis (equal); investigation (lead); validation (equal); visualization (equal); writing – original draft (lead). **Xiaogang Mao:** Data curation (supporting); formal analysis (supporting); investigation (supporting); resources (supporting). **Lin Li:** Data curation (supporting); formal analysis (supporting); software (supporting); validation (equal); visualization (equal). **Jiang Yang:** Funding acquisition (equal); investigation (equal); validation (equal); visualization (equal). **Hui Xing:** Conceptualization (supporting); funding acquisition (lead); methodology (supporting); resources (lead); supervision (equal). **Chunfan Jiang:** Conceptualization (lead); data curation (equal); formal analysis (equal); investigation (equal); project administration (lead); writing – review and editing (equal).

## FUNDING INFORMATION

National Natural Science Foundation of China (No. 81972449) and Foundation of Hubei University of Arts and Science (XK2019046) to Hui Xing.

## CONFLICT OF INTEREST STATEMENT

The authors declare that no conflicts of interest exist.

## ETHICS STATEMENT

This study was approved by the Medicine Ethics Committee of Xiangyang Central Hospital (Approval Code: 2022‐028) and was carried out in accordance with the Declaration of Helsinki.

## CONSENT TO PARTICIPATE

Written informed consent was obtained from individual or participants or their guardians.

## Supporting information


Appendix S1.

## Data Availability

All data generated or analyzed during this study are included in this published article.
