# Supplementary material for: High TPX2 expression results in poor prognosis, and Sp1 mediates the coupling of the CX3CR1/CXCL10 chemokine pathway to the PI3K/Akt pathway through targeted inhibition of TPX2 in endometrial cancer
Source: Cancer Med. 2024 Mar 11;13(5):e6958. doi: 10.1002/cam4.6958 (PMC10926884; doi:10.1002/cam4.6958)
Supplement: Supplementary file 1 — Appendix S1. [file CAM4-13-e6958-s001.docx]

**Supplementary table 1** RT-qPCR primer sequences

| Gene name | Forward primer | Reverse primer |
| --- | --- | --- |
| TPX2 | ACTTCCGCACAGATGAGCG | GGATGCTTTCGTAGTTCAGATGT |
| Sp1 | TGGCAGCAGTACCAATGGC | CCAGGTAGTCCTGTCAGAACTT |
| CXCL10 | CCACGTGTTGAGATCATTGCTAC | CTGCATCGATTTTGCTCCCC |
| CX3CR1 | AGTGTCACCGACATTTACCTCC | AAGGCGGTAGTGAATTTGCAC |
| GAPDH | GAGAAGGCTGGGGCTCATTT | TAAGCAGTTGGTGGTGCAGG |

**Supplementary table 2** Antibodies used in Western blotting

| Antibody names | Manufacturer | Cat.No. | Species | Dilution ratio |
| --- | --- | --- | --- | --- |
| TPX2 | Proteintech | 11741-1-AP | rabbit | 1:2000 |
| CX3CR1 | Proteintech | 13885-1-AP | rabbit | 1:2000 |
| CXCL10 | Proteintech | 10937-1-AP | rabbit | 1:2000 |
| Akt | abcam | ab152157 | rabbit | 1:2000 |
| p-Akt | Proteintech | 66444-1-Ig | mouse | 1:2000 |
| Sp1 | Proteintech | 21962-1-AP | rabbit | 1:5000 |
| GAPDH | abcam | ab8245 | mouse | 1:6000 |
| HRP- anti Rabbit | KPL | 074-1506 | goat | 1:5000 |
| HRP- anti Mouse | KPL | 074-1806 | goat | 1:5000 |

**Supplementary table 3** Clinical characteristics of patients with EC in TCGA

| Characteristics | levels | Overall |
| --- | --- | --- |
| n |  | 552 |
| Clinical stage, n (%) | Stage I | 342 (62%) |
|  | Stage II | 51 (9.2%) |
|  | Stage III | 130 (23.6%) |
|  | Stage IV | 29 (5.3%) |
| Primary therapy outcome, n (%) | PD | 20 (4.2%) |
|  | SD | 6 (1.2%) |
|  | PR | 12 (2.5%) |
|  | CR | 442 (92.1%) |
| Race, n (%) | Asian | 20 (3.9%) |
|  | Black or African American | 108 (21.3%) |
|  | White | 379 (74.8%) |
| Age, n (%) | <=60 | 206 (37.5%) |
|  | >60 | 343 (62.5%) |
| Weight, n (%) | <=80 | 243 (46%) |
|  | >80 | 285 (54%) |
| Height, n (%) | <=160 | 247 (47.2%) |
|  | >160 | 276 (52.8%) |
| BMI, n (%) | <=30 | 212 (40.8%) |
|  | >30 | 307 (59.2%) |
| Histological type, n (%) | Endometrioid | 410 (74.3%) |
|  | Mixed | 24 (4.3%) |
|  | Serous | 118 (21.4%) |
| Residual tumor, n (%) | R0 | 375 (90.8%) |
|  | R1 | 22 (5.3%) |
|  | R2 | 16 (3.9%) |
| Histologic grade, n (%) | G1 | 98 (18.1%) |
|  | G2 | 120 (22.2%) |
|  | G3 | 323 (59.7%) |
| Tumor invasion(%), n (%) | <50 | 259 (54.6%) |
|  | >=50 | 215 (45.4%) |
| Menopause status, n (%) | Pre | 35 (6.9%) |
|  | Peri | 17 (3.4%) |
|  | Post | 454 (89.7%) |
| Hormones therapy, n (%) | No | 297 (86.3%) |
|  | Yes | 47 (13.7%) |
| Diabetes, n (%) | No | 328 (72.7%) |
|  | Yes | 123 (27.3%) |
| Radiation therapy, n (%) | No | 279 (52.9%) |
|  | Yes | 248 (47.1%) |
| Surgical approach, n (%) | Minimally Invasive | 208 (39.2%) |
|  | open | 322 (60.8%) |
| OS event, n (%) | Alive | 458 (83%) |
|  | Dead | 94 (17%) |
| Age, median (IQR) |  | 64 (57, 71) |

**Supplementary table 4** Univariate and multivariate Cox regression analysis of the clinical characteristics associated with overall survival

**Supplementary table 5** Clinicopathological parameters of 609 patients with EC

| Characteristics | Levels | n(%) |
| --- | --- | --- |
| n |  | 609 |
| Age, mean ± SD | 29-83 | 55.31±8.63 |
| Age | <=60 | 441（72.41%） |
|  | >60 | 168（27.59%） |
| Histological type | Endometrioid | 565（92.78%） |
|  | Clear cell | 18（2.96%） |
|  | Serous | 26（4.26%） |
| Clinical stage | I | 418（68.64%） |
|  | II | 122（20.03%） |
|  | III | 65（10.67%） |
|  | IV | 4（0.66%） |
| Histological grade | G1 | 336（55.17%） |
|  | G2 | 167（27.42%） |
|  | G3 | 106（17.41%） |
| Tumor invasion(%) | <50 | 469（77.01%） |
|  | >=50 | 140（22.99%） |
| P53 | mutant | 132（21.67%） |
|  | wild | 477（78.33%） |
| Ki67proliferation index(%) | <50 | 474（77.83%） |
|  | >=50 | 135（22.17%） |
| Lymph node metastasis | Yes | 61（10.02%） |
|  | No | 319（52.38%） |
|  | No lymph node cleaning | 229（37.60%） |

**Supplementary table 6** The expression of TPX2 in EC and its correlation with the clinicopathological parameters

| Features | | Cases | Positive cases | Positive rate (%) | Strong positive cases | Strong positive rate (%) | P value  (positive) | P value  (strong positive) |
| --- | --- | --- | --- | --- | --- | --- | --- | --- |
| EC types | Ⅰ | 565 | 488 | 86.37 | 212 | 37.52 | 0.003 | 0.334 |
|  | Ⅱ | 44 | 30 | 68.18 | 20 | 45.45 |  |  |
| Histological  type | Endometrial | 565 | 488 | 86.37 | 212 | 37.52 | 0.001 | 0.225 |
|  | Serous | 26 | 20 | 76.92 | 14 | 53.85 |  |  |
|  | Clear cell | 18 | 10 | 55.56 | 6 | 33.33 |  |  |
| Tumor invasion(%) | <50 | 469 | 394 | 84.01 | 160 | 34.12 | 0.224 | <0.001 |
|  | >=50 | 140 | 124 | 88.57 | 72 | 51.43 |  |  |
| Clinical stage | Ⅰ | 418 | 342 | 81.82 | 102 | 24.40 | 0.001 | <0.001 |
|  | Ⅱ | 122 | 116 | 95.08 | 78 | 63.93 |  |  |
|  | Ⅲ+Ⅳ | 69 | 60 | 86.96 | 52 | 75.36 |  |  |
| Histological  grade | G1 | 336 | 292 | 86.90 | 120 | 35.71 | 0.155 | 0.101 |
|  | G2 | 167 | 141 | 84.43 | 62 | 37.13 |  |  |
|  | G3 | 106 | 84 | 79.25 | 50 | 47.17 |  |  |
| Lymph node metastasis | No | 319 | 268 | 84.01 | 100 | 31.35 | 0.729 | <0.001 |
|  | Yes | 61 | 52 | 85.25 | 40 | 65.57 |  |  |
|  | No lymph node  cleaning | 229 | 198 | 86.46 | 92 | 40.17 |  |  |
| P53 | Mutant | 132 | 108 | 81.82 | 48 | 36.36 | 0.053 | 0.686 |
|  | Wild type | 477 | 350 | 73.38 | 184 | 38.57 |  |  |
| Ki67 proliferation index(%) | >=50 | 135 | 112 | 82.96 | 54 | 40.00 | 0.494 | 0.617 |
|  | <50 | 474 | 406 | 85.65 | 178 | 37.55 |  |  |

**Supplementary table 7** Cox univariate and multivariate regression analysis with clinical characteristics.

| Characteristics | Total(N) | Univariate analysis | | Multivariate analysis | |
| --- | --- | --- | --- | --- | --- |
|  |  | Hazard ratio (95% CI) | P value | Hazard ratio (95% CI) | P value |
| TPX2 | 609 |  |  |  |  |
| Negative | 91 | Reference |  |  |  |
| Positive | 518 | 1.566 (1.178-2.081) | **0.002** | 1.211 (0.909-1.613) | 0.092 |
| Age | 609 |  |  |  |  |
| <=60 | 441 | Reference |  |  |  |
| >60 | 168 | 3.335 (2.022-5.500) | **<0.001** | 1.750 (0.982-3.120) | 0.058 |
| Histological type | 609 |  |  |  |  |
| Endometrioid | 565 | Reference |  |  |  |
| CCC | 18 | 18.295 (9.378-35.690) | **<0.001** | 2.829 (0.951-8.412) | 0.061 |
| Serous | 26 | 7.560 (4.158-13.748) | **<0.001** | 2.187 (0.888-5.387) | 0.089 |
| Clinical stage | 609 |  |  |  |  |
| I-II | 540 | Reference |  |  |  |
| III-IV | 69 | 3.823 (2.933-4.981) | **<0.001** | 2.055 (1.286-3.283) | **0.003** |
| Histological grade | 609 |  |  |  |  |
| G1 | 336 | Reference |  |  |  |
| G2+G3 | 273 | 2.844 (2.086-3.877) | **<0.001** | 0.932 (0.584-1.486) | 0.076 |
| Tumor invasion(%) | 609 |  |  |  |  |
| <50 | 469 | Reference |  |  |  |
| >=50 | 140 | 4.142 (2.510-6.833) | **<0.001** | 0.351 (0.147-0.838) | **0.018** |
| Ki67 proliferation index(%) | 609 |  |  |  |  |
| <50 | 474 | Reference |  |  |  |
| >=50 | 135 | 2.270 (1.363-3.783) | **0.002** | 1.074 (0.599-1.924) | 0.812 |
| Lymph node metastasis | 609 |  |  |  |  |
| No | 319 | Reference |  |  |  |
| No lymph node  cleaning | 229 | 0.478 (0.201-1.135) | 0.094 | 0.437 (0.180-1.058) | 0.066 |
| Yes | 61 | 10.138 (5.841-17.599) | **<0.001** | 3.624 (1.497-8.773) | **0.004** |
| P53 | 609 |  |  |  |  |
| Wild type | 477 | Reference |  |  |  |
| Mutant | 132 | 1.943 (1.168-3.232) | 0.01 | 1.030 (0.996-1.065) | 0.085 |


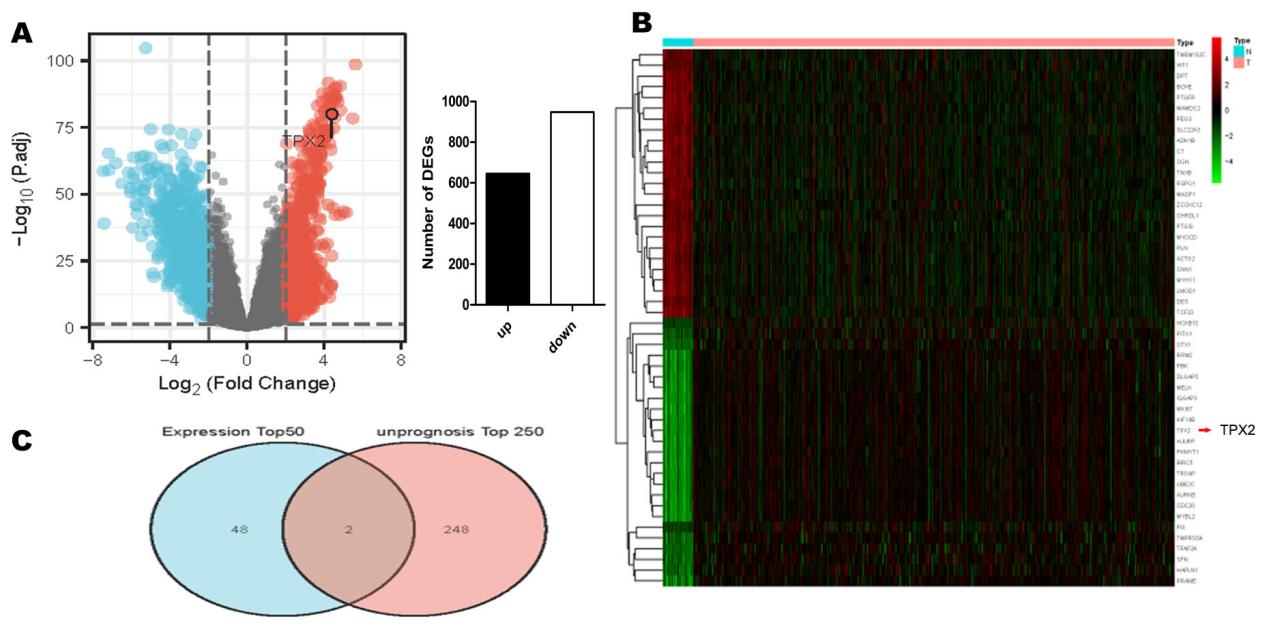


**Supplementary figure 1**  Screening of the key differentially expressed genes with TCGA dataset

(A) Differentially expressed genes (down-regulated genes denoted by blue, up-regulated genes denoted by red) in EC and normal adjacent tissues visualized with a volcano plot according to the threshold of ∣log_2_FC∣＞2 and adjusted *P* value＜0.05. The number of differentially expressed genes presented with a histogram. (B) The top 50 of differentially expressed genes in EC and normal adjacent tissues were visualized with a heatmap. (C) The intersection of the top 50 up-regulated differentially expressed genes from the TCGA database were compared with the top 250 genes with poor prognosis from HPA.


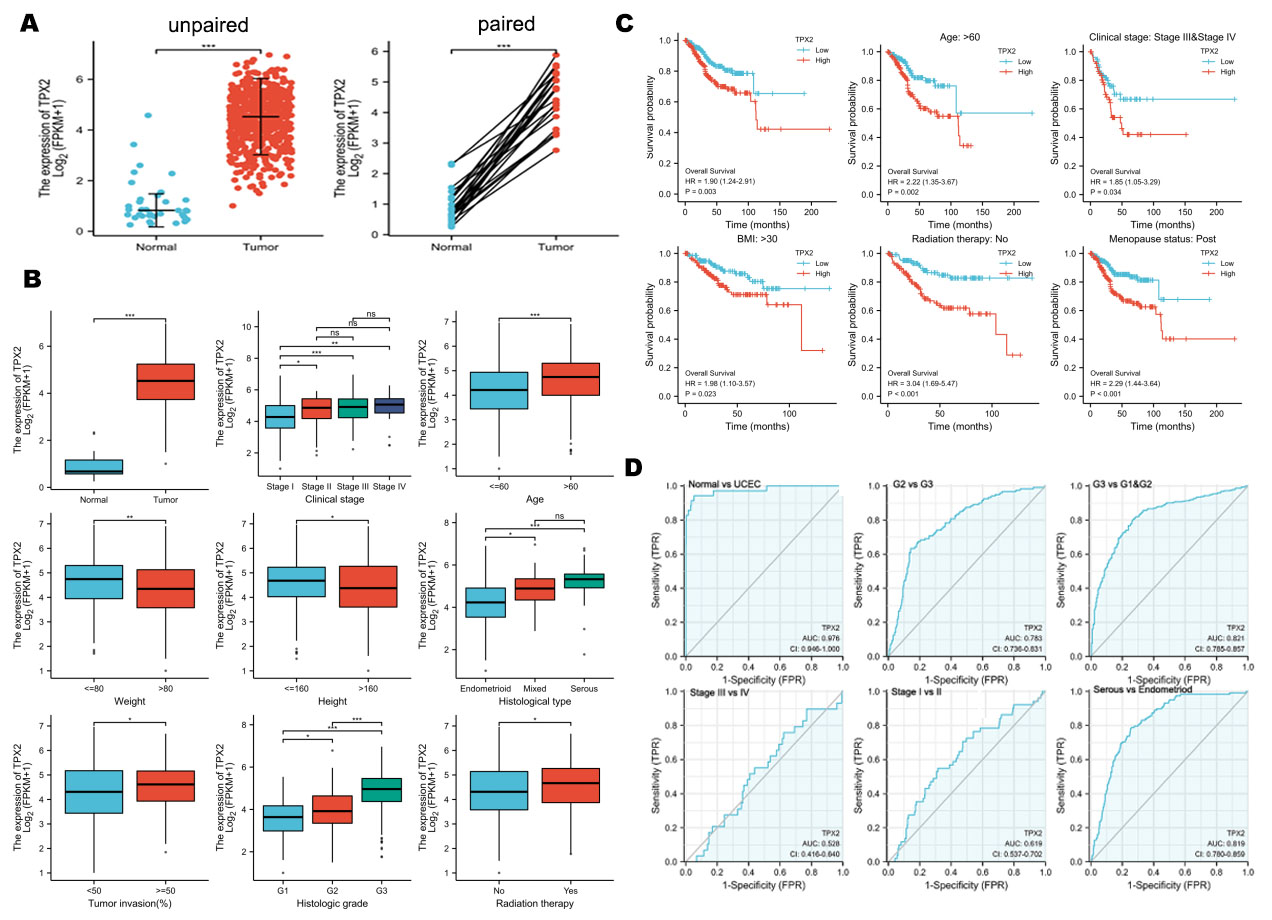


**Supplementary figure 2** The relationship between expression of TPX2 and the clinicopathological significance with TCGA dataset

1. Significant differences of TPX2 expression between tumor and normal tissues in the paired and unpaired groups displayed with dot plots. (B) The relationship between TPX2 expression and different clinicopathological parameters visualized with box-whisker plots. (C) Overall survival and subgroup analysis presented with Kaplan-Meier survival curve. (D) Diagnostic value of TPX2 expression between tumor and normal tissues as well as subgroup comparisons with ROC curve. * *p*＜0.05; ***p*＜0.01; *** *p*＜0.001; ns, no significance.


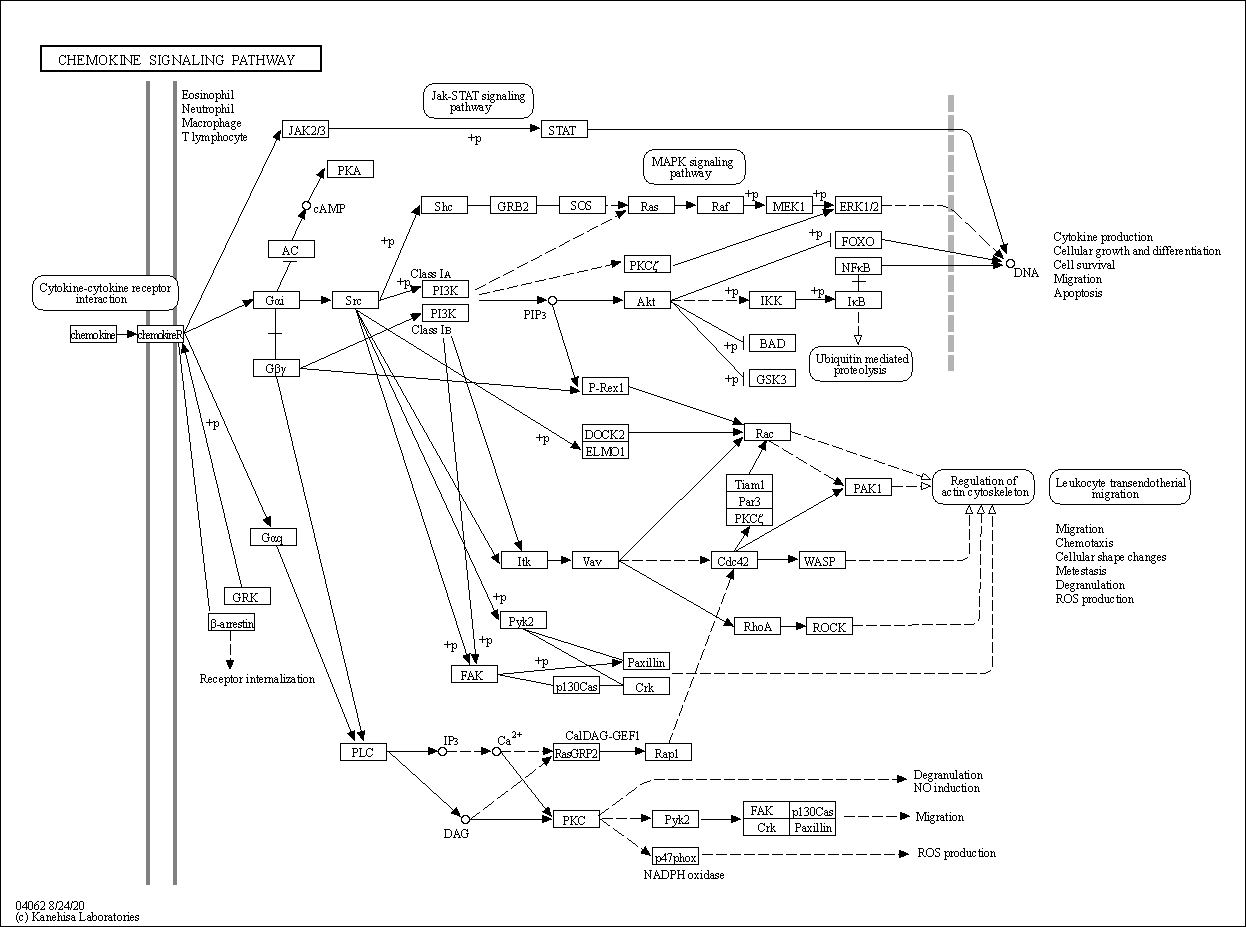


**Supplementary figure 3**  Chemokine pathway is coupled to PI3K-Akt pathway


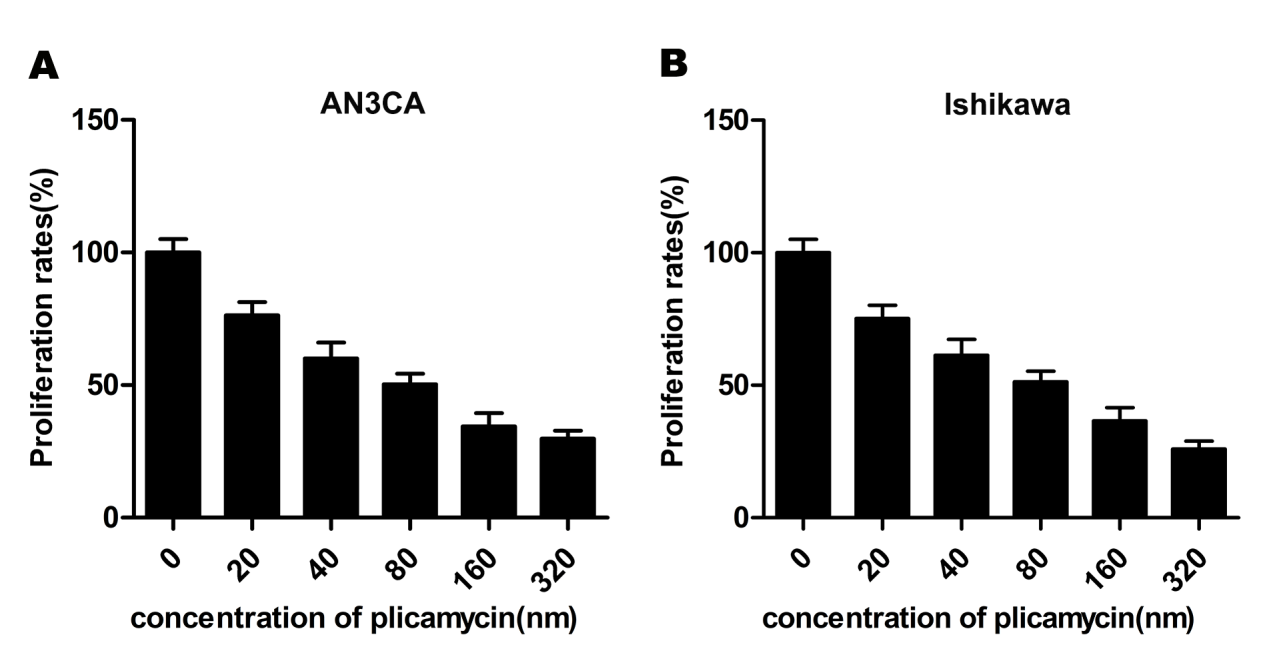


**Supplementary figure 4** The Sp1 inhibitor, Plicamycin with a concentration of 80nM, demonstrates the ability to achieve 50% inhibition of SP1 activity. (A) AN3CA cell line and (B) Ishikawa cell line.


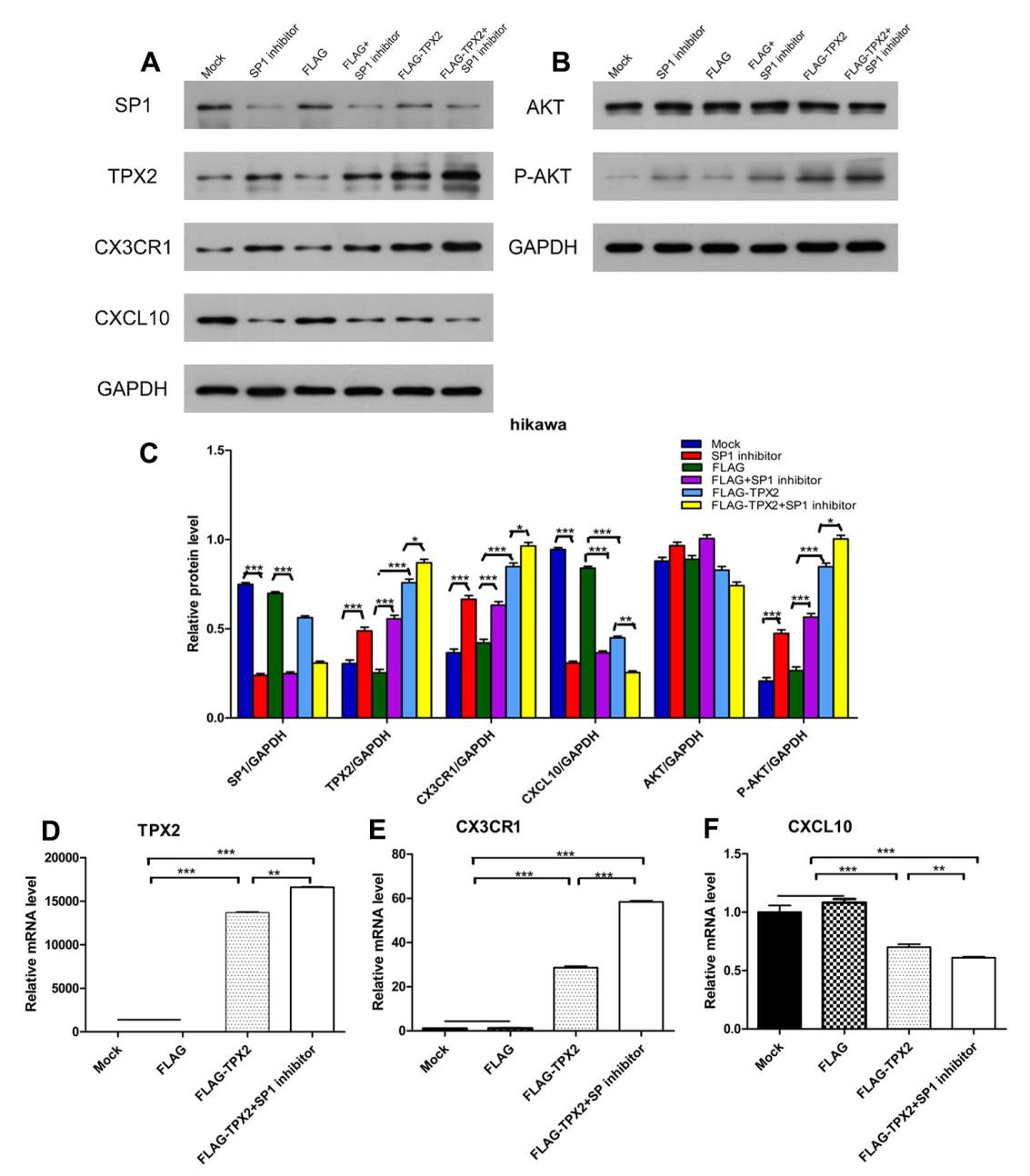


**Supplementary figure 5** Co-transfection combination experiment in Ishikawa cells

(A, B) Sp1 inhibitor, FLAG, FLAG-TPX2, and Sp1 inhibitor + FLAG-TPX2 were transfected into Ishikawa cells, and the protein expression levels of related molecules such as Sp1, TPX2, CX3CR1, CXCL10, Akt, and p-Akt were detected in Ishikawa cells with Western blotting and quantitative presentation with histogram (C); (D, E, F) FLAG, FLAG-TPX2, and Sp1 inhibitor + FLAG-TPX2 were transfected into Ishikawa cells, and the mRNA expression levels of related molecules such as Sp1, TPX2, CX3CR1, and CXCL10 were detected with RT-qPCR.
